# A reduction in CD90 (THY-1) expression results in increased differentiation of mesenchymal stromal cells

**DOI:** 10.1186/s13287-016-0359-3

**Published:** 2016-07-28

**Authors:** Daniela A. Moraes, Tatiana T. Sibov, Lorena F. Pavon, Paula Q. Alvim, Raphael S. Bonadio, Jaqueline R. Da Silva, Aline Pic-Taylor, Orlando A. Toledo, Luciana C. Marti, Ricardo B. Azevedo, Daniela M. Oliveira

**Affiliations:** 1 Departamento de Genética e Morfologia, Universidade de Brasília, Brasília, DF Brazil; 2 Departamento de Ciências da Saúde, Universidade de Brasília, Brasília, DF Brazil; 3 Departamento de Neurologia e Neurocirurgia, Universidade Federal de São Paulo, São Paulo, SP Brazil; 4 Hospital Israelita Albert Einstein, Instituto de Ensino e Pesquisa - Centro de Pesquisa Experimental São Paulo, São Paulo, SP Brazil; 5 IB-Departamento de Genética e Morfologia, Universidade de Brasília - UNB, Campus Universitário Darcy Ribeiro, Asa Norte, Brasília, CEP 70910-970 Brazil; 6 Centro Universitario do Distrito Federal UDF, Brasília, DF Brazil

**Keywords:** Mesenchymal stem cells, Mesenchymal stromal cells, CD90, Thy-1, Fibroblast, Differentiation

## Abstract

**Background:**

Mesenchymal stromal cells (MSCs) are multipotent progenitor cells used in several cell therapies. MSCs are characterized by the expression of CD73, CD90, and CD105 cell markers, and the absence of CD34, CD45, CD11a, CD19, and HLA-DR cell markers. CD90 is a glycoprotein present in the MSC membranes and also in adult cells and cancer stem cells. The role of CD90 in MSCs remains unknown. Here, we sought to analyse the role that CD90 plays in the characteristic properties of in vitro expanded human MSCs.

**Methods:**

We investigated the function of CD90 with regard to morphology, proliferation rate, suppression of T-cell proliferation, and osteogenic/adipogenic differentiation of MSCs by reducing the expression of this marker using CD90-target small hairpin RNA lentiviral vectors.

**Results:**

The present study shows that a reduction in CD90 expression enhances the osteogenic and adipogenic differentiation of MSCs in vitro and, unexpectedly, causes a decrease in CD44 and CD166 expression.

**Conclusion:**

Our study suggests that CD90 controls the differentiation of MSCs by acting as an obstacle in the pathway of differentiation commitment. This may be overcome in the presence of the correct differentiation stimuli, supporting the idea that CD90 level manipulation may lead to more efficient differentiation rates in vitro.

**Electronic supplementary material:**

The online version of this article (doi:10.1186/s13287-016-0359-3) contains supplementary material, which is available to authorized users.

## Background

Mesenchymal stromal cells (MSCs) are multipotent progenitor cells identified by their plastic-adherence when maintained under standard culture conditions, self-renewability, and differentiation into several mesodermal lineages [1–3]. MSCs are classically able to differentiate into osteoblasts, adipocytes, and chondroblasts in vitro [4]. Since their initial description as colony-forming cell units present in the bone marrow [5], MSCs have been isolated from many tissue sources such as placenta [6], dental pulp [7], tendons [8], scalp tissue [9], adipose tissue [10], umbilical cord blood [11], umbilical cord perivascular cells [12], umbilical cord Wharton’s jelly [13], synovial membrane [2], amniotic fluid [14], and breast milk [15]. Due to their relatively easy isolation, multi-differentiation potential, low antigenicity, and good proliferation/expansion in cell culture, MSCs are considered ideal candidates for cell-based regenerative therapies [16]. Based on the minimal criteria established by the International Society for Cellular Therapy (ISCT), human MSCs are identified by a combination of high CD105, CD73, and CD90 expression, and very low/no CD34, CD45, CD11a, CD19, and HLA-DR expression [4, 17]. Currently, there is no unique cell marker capable of solely isolating and defining MSCs. The observation that only a subpopulation of plastic-adherence isolated MSCs show multipotency [18] has led to a search for an ideal and definitive single MSC marker that would not only be specific to MSC, but would allow direct correlation with stemness [19].

Although CD90 and STRO-1 are broadly used to identify MSCs, neither of them is specific to MSCs [20–22]. STRO-1 is only expressed in a low percentage of MSCs. Some authors also discuss the absence of this marker in MSCs from all tissue sources [12, 19, 23], and it remains unclear, in the current literature, whether STRO-1 expression correlates to MSC stemness. On the other hand, CD90 is highly expressed in all MSCs, irrespective of the source, and it is a good marker for CFU-F enrichment [24]. High CD90 expression has also been related to the undifferentiated status of MSCs, since a decrease in CD90 level can be correlated with the temporal lineage commitment in vitro [25].

CD90, or Thy-1, is a 25–37 KDa glycosylphosphatidylinositol (GPI)-anchored glycoprotein [26]. CD90 was first detected in mice T cells [27] and later found to be expressed in thymocytes, T cells, neurons, hematopoietic stem cells, cancer stem cells, endothelial cells, and fibroblasts [28]. Although it has been shown that CD90 is conserved among different species, its function seems to vary according to cell type [29]. CD90 has been reported to participate in T-cell activation [30], neuritis outgrowth modulation [31], vesicular release of neurotransmitter at the synapse [32], astrocyte adhesion [33], apoptosis in carcinoma cells [34], tumour suppression [35–37], wound healing [38], fibrosis [39, 40], and fibrogenesis [41]. Furthermore, it regulates fibroblast focal adhesion, cytoskeleton organization, and cell migration [42]. In mouse models, activation of CD90 expression can be observed in inflammation, wound healing, and tumour development [43]. Recent studies suggest that CD90 has a role in oncogenesis, and it has also been proposed as a marker for cancer stem cells (CSCs) in various malignancies [44–51].

Despite an increasing number of studies suggesting CD90 participation in MSC self-renewal and differentiation [52], its function in MSC biology remains unknown. The unveiling of the function of CD90 in MSCs may further facilitate the in vitro manipulation of MSCs and consequently MSC-based therapies for regenerative medicine. In this study, we investigated the function of CD90 in MSC biology. To achieve this objective, we analysed the effect of CD90 knockdown on proliferation, morphology, and differentiation of human MSCs.

## Methods

### Subjects and cell culture

The cells were obtained with the approval of the Ethics Committee of the Faculty of Health Sciences at the University of Brasilia (Brazil) and University of São Paulo (Brazil). MSCs were isolated from healthy human tissues and cultured as previously reported. In the present study, we obtained MSCs from three different tissue sources: dental pulp [7] (three donors), adipose tissue [10] (two donors), and amniotic fluid [14] (two donors). After isolation, cells were cryopreserved and stored in liquid nitrogen. For the assays we used cells that were stored for no longer than 1 year. Briefly, cells were thawed and expanded in a regular medium of Dulbecco’s modified Eagle’s medium (DMEM-LG; Sigma Chemical), supplemented with 10 % fetal bovine serum (FBS; Gibco), 100 units/ml penicillin, 100 mg/ml streptomycin (Gibco), and 10 μl/ml l-glutamine (Gibco) at 37 °C, 5 % CO_2_ [1]. The medium was changed every 48 h.

### Lentiviral transduction for CD90 depletion

For lentiviral transduction, MSC isolates (a total of seven samples at cell passage 2) were cultured in a 75-cm^2^ flask in medium containing 10 % FBS (Gibco), 100 units/ml penicillin, 100 mg/ml streptomycin (Gibco), 10 μl/ml l-glutamin (Gibco) at 37 °C, 5 % CO_2_. When cells reached a confluence of 60 %, transduction was performed in the presence of 8 μg/ml Polybrene (Sigma-Aldrich) according to the manufacturer’s instructions (Santa Cruz Biotechnology). CD90 small hairpin (sh)RNA-expressing lentivirus (shRNA CD90) or non-targeting shRNA-expressing scramble sequences of RNA (shRNA control) were then added to the cells at a multiplicity of infection (MOI) of 10. The medium was changed after 24 h. Three days after transduction, stable clones of MSCs expressing CD90-shRNA (shRNA CD90 MSC) and control shRNA (shRNA control MSC) were selected using 5 μg/ml Puromycin (Sigma-Aldrich) for 10 days. The medium was changed every 48 h.

### Real-time quantitative PCR

Total RNA was extracted from MSCs using Illustra RNAspin Mini (GE Healthcare), according to the manufacturer's guidelines. cDNA was prepared with High-Capacity cDNA Reverse Transcription (Applied Biosystems) and used as templates for polymerase chain reaction (PCR). The Kit Power Up SYBR Green Master Mix (Applied Biosystems) was used to quantify CD90 gene expression by quantitative real-time (qRT)-PCR under conditions recommended by the manufacturer and using the following primers: CACCCTCTCCGCACACCT (forward) and CCCCACCATCCCACTACC (reverse). For normalization of the data, the housekeeping gene glyceraldehyde 3-phosphate dehydrogenase (GAPDH) mRNA was used (forward primer: AGAAGGCTGGGGCTCATTTG; reverse primer: AGGGGCCATCCACAGTCTTC). qRT-PCR was performed with the StepOne Plus Real-Time PCR System. A standard curve was generated for each primer pair, and genes of interest were assigned a relative expression value interpolated from the standard curve using the threshold cycle. All expression values were normalized against GAPDH. All amplifications were done in triplicate.

### Magnetic separation of the MSCs for negative selection of CD90

Cell purification was performed according to the manufacturer’s instructions (MiltenyiBiotec). To isolate the CD90-negative MSC population, shRNA CD90 MSCs were incubated with anti-CD90-coupled magnetic beads (MiltenyiBiotec, Germany) for 15 min at 4 °C, rinsed, and placed in a column. The negative fraction (CD90-negative MSCs) was collected, and cell purity checked by flow cytometry (FACSVERSE-BD Biosciences, San Jose, CA, USA) and FlowJo analysis software (TreeStar, Ashland, OR, USA).

### Flow-cytometric analysis

Commercially available monoclonal antibodies were used for MSC immunophenotyping following the manufacturer’s instructions. Subcultures at passage 3 were used for the flow-cytometric analysis. MSCs were lifted using TrypLE (Invitrogen, Carlsbad, CA, USA) and centrifuged for 5 min at 1000 rpm. The supernatant was discarded by aspiration and the cells incubated for 30 min in a dark environment in a flow cytometry buffer (phosphate-buffered saline (PBS), 2 % FBS) containing monoclonal antibodies against cell surface molecules and their respective isotype controls. The following antibodies were used: CD14-FITC; CD29-PE; CD31-PE; CD34-PE; CD44-PE; CD73-PE; CD90-APC; CD90-FITC; CD106-FITC; CD166-PE and CD166-PerPC-Cy5.5; CD45-PerCP-Cy5.5; HLA-DR-PerCP-Cy5.5 (Biosciences); and CD105-PE (clone 8E11; Chemicon, Temecula, CA, USA). Mouse IgG1-FITC, IgG1-PE, IgG1 PerCP-Cy5.5, IgG1-APC (Biosciences), and IgG2A-FITC (AbDSerotec, UK) were used as isotype controls. Cells were analysed using a fluorescence-activated cell sorter (CyFlowSpace-Partec, Germany; FACSVERSE-BD or FACSARIA-BD, both from BD Biosciences) and the data analysed using FlowJo analysis software (TreeStar).

### MSC morphology analysis

Transduced and non-transduced MSCs at passage 3 were placed, in triplicate, in 24-well culture plates (5 × 10^4^ cells/well). After cell concentration reached a confluence of 70 %, media were removed and the cells were washed with PBS and fixed with a 4 % paraformaldehyde solution for 15 min at room temperature. Cells were then washed with PBS, stained with Kit Instant Prov (NewProv, Brazil) and rewashed with PBS. Cell morphology (shape and size) was then analysed under an Axiovert inverted microscope (Zeiss, Germany) and EVOS FL cell imaging system (Life Technologies, Eugene, OR, USA).

### Growth assay

For the assessment of growth characteristics, MSCs (1 × 10^5^ cells, at passage 3 after transduction, passage 5 after isolation) were seeded in 75-cm^2^ culture flasks in MSC culture medium. Every 48 h, three replicate flasks were trypsinised and viable cells counted with a haemocytometer. MSC viability was evaluated by Trypan blue exclusion assay.

### Lymphocyte proliferation assay

Peripheral blood mononuclear cells were isolated from peripheral blood and separated using the standard method with Ficoll-Paque PLUS (Amersham Biosciences, Uppsala, Sweden). The mononuclear cells were washed twice with PBS buffer. Cells were then counted in an automated cell counter (2.0 Scepter, Millipore), resuspended to a final concentration of 10^4^ cells/ml and labelled with CFSE (Sigma-Aldrich). The CFSE was adjusted to a final concentration of 5 μM and incubated for 10 min at 37 °C. The reaction was stopped by adding RPMI with 10 % FBS. In immediate succession, 2 × 10^4^ lymphocytes were cultured with or without 5 × 10^4^ MSCs previously adhered to the bottom of a 24-well plate in a total volume of 1 ml per well of RPMI with 10 % FBS medium. To evaluate the lymphocyte proliferation rate in the presence of MSCs, cell suspensions were activated with a phytohaemagglutinin (PHA; Sigma, USA) stimulus at a final concentration of 1 μg/ml in cell culture and maintained at 37 °C with 5 % CO_2_ for 5 days for subsequent assessment by flow cytometry (CyFlowSpace-Partec, Germany) and the FlowJo analysis software (TreeStar) [53]. Suspension cells were stained with CD8-PE antibody (Biosciences), and lymphocyte proliferation was measured according to CFSE staining on gated population.

### In vitro differentiation assays

To evaluate the differentiation potential of MSCs, cells were subjected to in vitro osteogenic and adipogenic differentiation according to the established protocols [1]. Transduced and non-transduced MSCs at passage 4 (passage 2 after transduction) were seeded in 24-well plates at a density of 5 × 10^4^ cells/well. When a confluence of 80 % was achieved, the regular medium was replaced with an induction medium, which was refreshed every 72 h for 21 days. Cells cultured in regular medium were used as controls.

#### Osteogenic differentiation

MSCs were placed in 24-well plates at a density of 5 × 10^4^cells/cm^2^ the previous day and then treated with osteogenic supplements as previously described [1] for 21 days. The osteogenic medium contained 100 nM dexamethasone (Sigma-Aldrich), 10 mM 2-β-glycero-phosphate (Sigma-Aldrich), and 50 μM of l-ascorbic acid-2-phosphate (Sigma-Aldrich). Osteogenic differentiation was evaluated by alkaline phosphatase (ALP) activity, calcium concentration determination, and colorimetric Alizarin red staining. Mineralized matrix formation after osteogenic differentiation was detected as previously described [1]. Samples were fixed with 4 % paraformaldehyde for 15 min, rinsed in PBS, and dyed for 20 min with 40 mM Alizarin Red solution (Sigma-Aldrich) at pH 4.2 and room temperature. Cells were washed five times with distilled water, followed by an immediate 15-min rinse with PBS to reduce non-specific dying. The resulting samples were analysed and photographed under an Axiovert inverted microscope (Zeiss, Germany). To determine alizarin red concentration, the samples were exposed to 10 mM sodium phosphate containing 10 % cetylpyridinium chloride (Sigma-Aldrich) at pH of 7.0 for 15 min at room temperature. The Alizarin Red concentration was determined by measuring absorption at 562 nm using a spectrophotometer (SpectraMax M2, Molecular Devices, USA). Results were expressed as a percentage of the respective controls, which were normalized to 100 % [54]. Lysate alkaline phosphatase activity was measured spectrophotometrically using a Sigmafast p-nitrophenyl phosphate kit (Sigma-Aldrich). For ALP assays, cells were washed with PBS and lysed in 0.05 % Triton X-100 through three cycles of freezing and thawing. A lysate aliquot was incubated with p-nitrophenyl phosphate substrate (p-NF) at 37 °C for 30 min. The reactions were stopped by adding 5 μl 1 N NaOH and absorbance measured at 405 nm using a spectrophotometer (SpectraMax M2, Molecular Devices) [55]. A pattern curve of p-NF was established in order to determine the enzymatic activity. Samples were normalized and total protein quantification determined by the Lowry method [56].

The quantitative levels of calcium in cell samples were determined for both osteogenic differentiation-induced and non-induced cells. Supernatant calcium concentration was determined by colorimetry using the ortho-cresolphthalein complexone (o-CPC) method [57]. Cells were trypsinised, resuspended in PBS, and then reacted with a calcium reagent containing 0.69 mol/l ethanolamine buffer, 0.2 % sodium azide, 0.338 mmol/l 0-cresolphthalein complexone, and 78 mmol/l 8-hydroxynquinoline-13. Cell reactions were read by a spectrophotometer (Advia2400, Siemens).

#### Adipogenic differentiation

For adipogenic induction, cells were seeded in 24-well plates at a density of 5 × 10^4^ cells/cm^2^. When the cells reached confluence, they were treated with an adipogenic induction medium containing 5 mg/ml insulin, 5 mmol indomethacin, 1 mmol dexamethasone, and 0.5 mmol/l isobutyl-1-methylxanthine (all from Sigma-Aldrich) in regular medium. Adipocyte formation was monitored by the appearance of lipid droplets under a microscope. After the induction period, cytochemical analysis of the differentiated and control cells was performed by conventional optical microscopy. The cells were fixed in 4 % formaldehyde for 15 min, rinsed in PBS, and dyed for 30 min with 0.5 % Oil Red O (Sigma-Aldrich) in ethanol. Cells were subsequently washed five times with distilled water to remove any excess dye. Quantification of lipid accumulation was achieved by extracting Oil Red-O from stained cells with isopropanol and measuring the OD of the extract at 510 nm using a Spectramax M2 spectrophotometer (Molecular Devices) [58].

### Statistical analysis

Statistical analysis was performed using the software GraphPad™ (San Diego, CA, USA). Quantitative data were expressed as mean ± standard deviations (SD) and statistical analyses of variance (ANOVA). Multiple comparisons were performed with Tukey's HSD test when appropriate. Findings with *p* < 0.05 were considered statistically significant.

## Results

### MSC isolates and purity

MSCs were obtained from dental pulp (DPSC; three donors), amniotic fluid (AF-MSC; two donors), and adipose tissue (ADSC; two donors). The success rate of isolating MSCs from all tissues was 100 %. Cells from all three sources (a total of seven isolates) contained a high number of adherent MSC-like cells which proliferated rapidly in number. Analysis of positive and negative characteristics for human MSC surface antigens by flow cytometry for cultured MSCs showed a high purity (≥97 %) of the cells (Additional file 1: Table S1).

### Analysis of the CD90 downregulated expression effect in MSCs

**Fig. 1:**
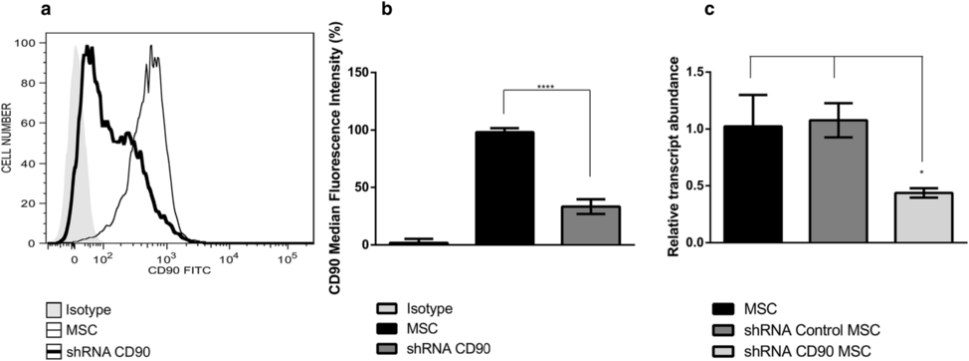
Reduction of CD90 in MSCs. **a** Non-transduced mesenchymal stromal cells (*MSC*) and MSCs transduced with lentiviral particles expressing short hairpin (sh)RNA against CD90 (*shRNA CD90 MSC*) were analysed by flow cytometry. An accentuated decrease in CD90 expression is observed in shRNA CD90 MSC (*thick line*), whereas non-transduced MSCs (*slim line*) expressed high levels of CD90. The *shaded* histogram indicates staining with isotype control antibody. Representative histograms from dental pulp MSCs are shown. **b** Significant decrease of CD90 median fluorescence intensity (MFI) on shRNA CD90 MSCs when compared to non-transduced MSCs. (MFI = MFI marker – MFI isotype). Bar graphs represent the average mean fluorescence intensity as the median ± SD of CD90-FITC on cell lines used in this work; *n* = 7; ****p < 0.001. **c** Relative mRNA expression levels of CD90 in MSC, shRNA control MSC, and shRNA CD90 MSC had consistently low expression of CD90. Data are presented as mean ± SD of experiments performed in triplicate; *p < 0.05

To initiate our study, we reduced CD90 expression in MSCs (DPSC, AF-MSC, and ADSC) by transducing commercially available lentiviruses expressing three CD90 shRNAs. After transduction, the MSC lines stably expressing shRNA CD90 and shRNA control were established by antibiotic selection. To confirm CD90 reduction, unmodified/non-transduced MSCs, shRNA CD90 MSCs, and shRNA control MSCs were analysed by flow cytometry (Fig. 1a and b) and qRT- PCR (Fig. 1c). Non-transduced MSCs and shRNA control MSCs showed the same level of CD90 expression (mean 98 %), whereas shRNA CD90 MSCs presented reduced CD90 expression (mean 23.9 %) (Fig. 1b). Transduction and establishment of shRNAs (CD90 and control) expressing MSCs were performed using samples from all tissues, with similar levels of reduction in CD90 expression observed in all samples (Additional file 1: Table S1). qRT- PCR confirmed that shRNA CD90 used here effectively reduced transcript levels of CD90 (Fig. 1c).

**Fig. 2:**
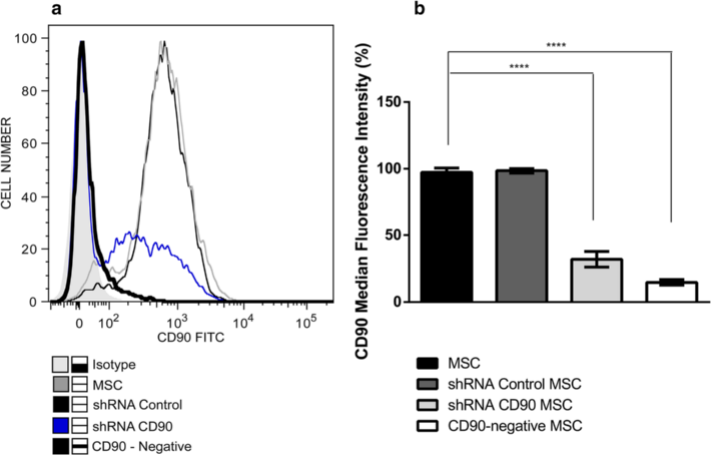
CD90-negative selection of shRNA CD90 MSCs by magnetic-activated cell sorting. Mesenchymal stromal cells (*MSCs*) expressing short hairpin (sh)RNA CD90 (*shRNA CD90 MSC*) were magnetically labelled with CD90 microbeads, and MSC populations negatively selected for CD90 (*CD90-negative MSCs*). **a** Histogram superposition: isotype control (*shaded histogram grey*), non-transduced MSCs (*black slim line*), shRNA control MSCs (*grey line*), shRNA CD90 MSCs (*blue line*), and CD90-negative MSCs (*black thick line*).Representative histograms from a dental pulp MSC group are shown. **b** Graph showing mean fluorescence intensity (MFI) of CD90 marker (MFI = MFI marker – MFI isotype) on cells; *n* = 7, independent experiments with MSCs derived from 7 tissue donors; ****p < 0.001

Since CD90 expression was not completely ablated in shRNA CD90 MSCs, we submitted shRNA CD90 MSC samples to magnetic-activated cell sorting and collected the post-separation CD90-negative fraction (subsequently termed CD90-negative MSCs). CD90-negative MSCs were characterized by flow cytometry to verify purification success. Flow cytometry analysis confirmed that the CD90-negative MSCs samples expressed lower levels of CD90 than shRNA CD90 MSCs (Fig. 2).

### Morphology and growth kinetics

**Fig. 3:**
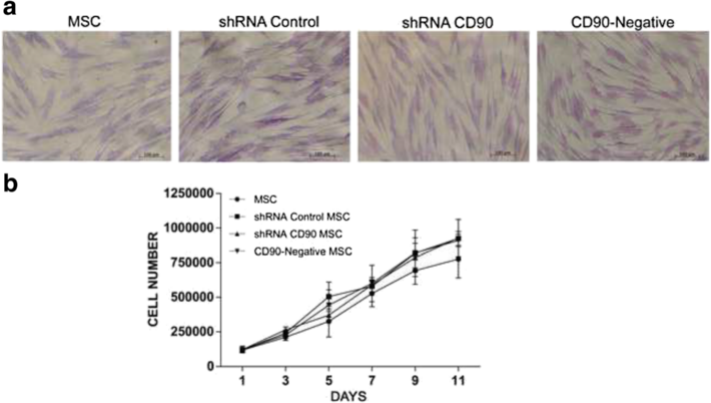
Reduction of CD90 expression does not affect mesenchymal stromal cell (*MSC*) morphology and proliferation rate. **a** Representative phase contrast microscopy images of MSCs derived from dental pulp. All MSCs displayed a spindle-like morphology exhibiting relatively thin processes extending from the cell bodies. The photographs shown here are representative of all samples analysed (*n* = 7). **b** Proliferation curves of non-transduced MSCs, short hairpin (sh)RNA control MSCs, shRNA CD90 MSCs, and CD90-negative MSCs. Data shown represent the mean ± SD of two independent experiments performed in triplicate with dental pulp MSCs from two tissue donors; *p < 0.05

In our cellular morphology analysis of the cells used in this study, we observed no differences in the shape and size of MSCs, shRNA control MSCs, shRNA CD90 MSCs, CD90-negative MSCs, and non-transduced MSCs (Fig. 3a). The shRNA CD90 MSCs and CD90-negative MSCs derived from all three sources displayed characteristic MSC/fibroblast-like morphology. We also observed that shRNA CD90 MSCs and CD90-negative MSCs maintained their capacity to form colonies for up to 10 passages, suggesting that CD90 is not involved in the maintenance of MSC cell morphology and colony-forming ability.

In order to assess the role of CD90 in MSC proliferation rate, cell growth curves for MSCs, shRNA control MSCs, shRNA CD90 MSCs, CD90-negative MSCs, and non-transduced MSCs at the same corresponding cell passage (cell passage 5) were conducted in parallel (Fig. 3b). Analysis of the area under the curve showed no significant difference in proliferation rates. The trypan blue exclusion assay also showed no difference in cell viability.

### Lymphocyte proliferation analysis

**Fig. 4:**
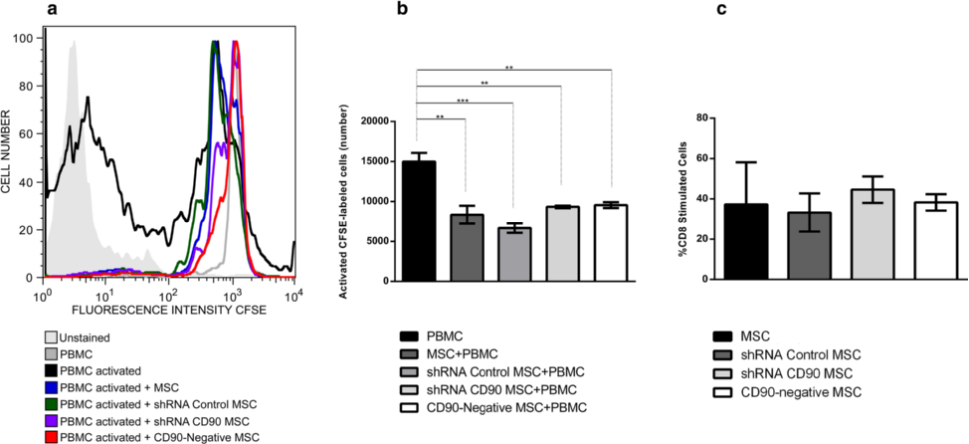
T-cell proliferation assays. Assays were performed using carboxyfluorescein succinimidyl ester (*CFSE*)-labelled human peripheral blood mononuclear cells (*PBMC*) activated with phytohaemagglutinin and co-cultured with or without human dental pulp mesenchymal stromal cells (*MSC*) for 5 days. **a** Representative histograms from dental pulp MSC cytometry analysis are shown (*n* = 3). **b** Histograms showing number of CFSE-labelled activated cells. **c** Histograms showing percentage proliferation of CFSE-labelled CD8+ T cells. *n* = 3; *p < 0.05. *sh* short hairpin

We also investigated whether CD90 expression in MSCs would affect the inhibitory effect of MSCs on non-specific mitogen-stimulated lymphocytes in an in vitro assay. The assay showed that shRNA CD90 MSCs and CD90-negative MSCs suppressed peripheral blood mononuclear cell proliferation to the same extent as MSCs and shRNA control MSCs and non-transduced MSCs (Fig. 4a and b), indicating that a reduction in the expression of CD90 does not affect the characteristic immunosuppressive effect of MSCs on lymphocyte proliferation. Further analysis shows that ablation of CD90 on MSCs also does not affect the percentage of proliferated CD8+ T cells (Fig. 4c).

### Flow cytometry immunophenotyping

**Fig. 5:**
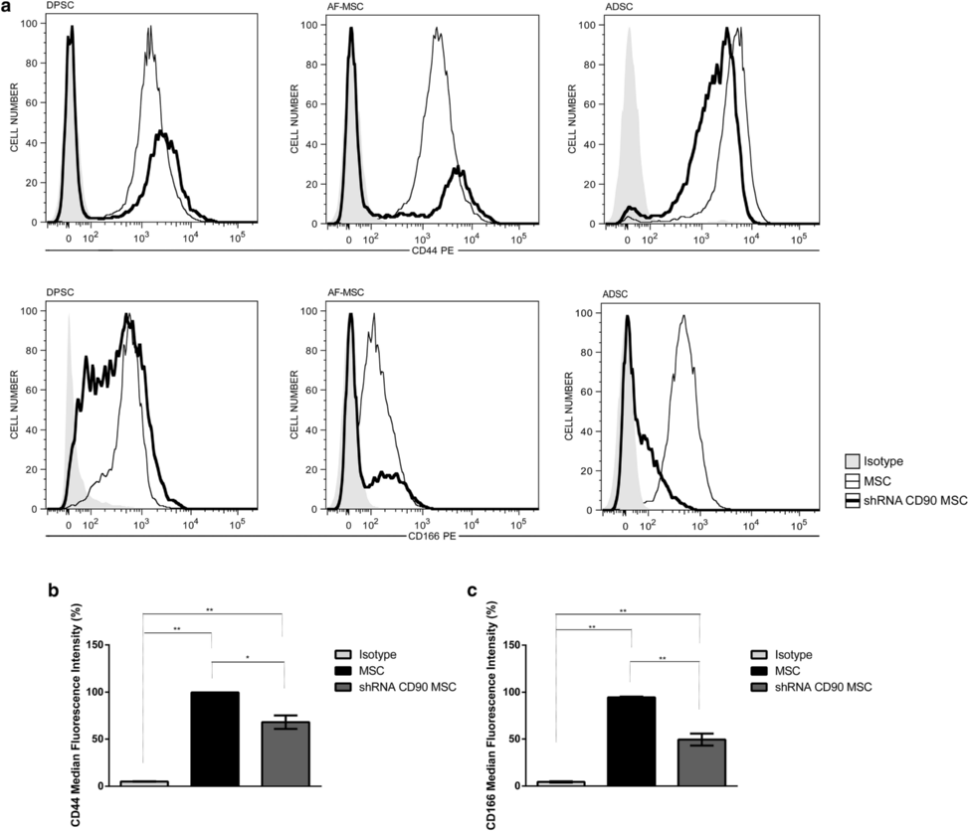
Reduction of CD90 expression leads to a reduction in the expression of CD44 and CD166 in mesenchymal stromal cells (*MSCs*). **a** MSCs (*slim line*) and shRNA CD90 MSCs (*thick line*) were analysed by flow cytometry to evaluate their expression of CD44 and CD166. The results showed a significant reduction of CD44 and CD166 expression in shRNA CD90 MSCs from different tissue sources. An isotype control (*shaded histogram*) was used to establish the boundary between negative and positive fluorescent regions. Median fluorescence intensities (MFI) of **b** CD44 and **c** CD166 markers on MSCs are shown (MFI = MFI marker – MFI isotype); *n* = 7; *p < 0.05. *ADSC* adipose tissue mesenchymal stromal cell, *AF-MSC* amniotic fluid mesenchymal stromal cell, *DPSC* dental pulp mesenchymal stromal cell, *sh* short hairpin

We further analysed the cell expression of the MSC marker panel. As expected, and as for non-transduced MSCs, shRNA control MSCs, shRNA CD90 MSCs, and CD90-negative MSCs were negative for the expression of the following markers: CD14, CD31, CD34, CD45, CD106, and HLA-DR, but they were positive for CD29, CD73, and CD105 (Additional file 1: Table S1 and Additional file 2: Figure S1). Surprisingly, we found a reduction in the expression of the CD44 and CD166 markers in shRNA CD90 MSCs, suggesting that the CD90 reduction is linked to the decrease in CD166 and CD44 expression (Fig. 5a and b). These reductions were observed in MSCs from all three sources (Fig. 5a).

### CD90 and MSC differentiation

**Fig. 6:**
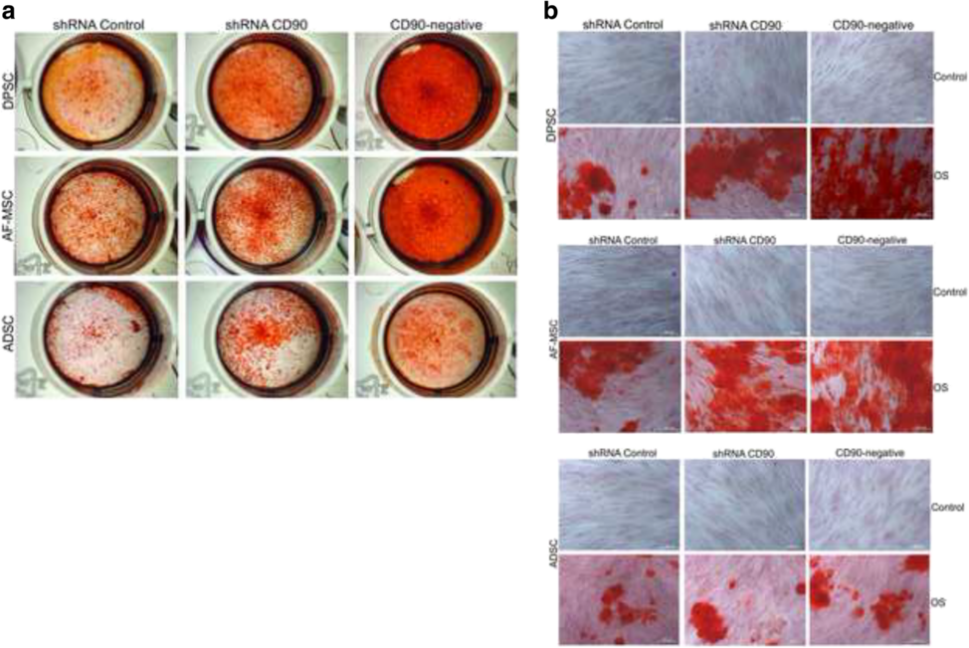
Reduction of CD90 expression stimulates MSC osteogenesis. MSCs, shRNA control MSCs, shRNA CD90 MSCs, and CD90-negative MSCs from dental pulp (*DPSC*), amniotic fluid (*AF-MSC*) and adipose tissue (*ADSC*) were tested in parallel for their ability to differentiate in vitro into osteogenic lineages. Calcified deposits were evidenced by Alizarin Red Staining (AR) in cells after 4 weeks of growth in osteogenic induction medium. Calcification was assessed by gross appearance (**a**) and light microscopy (**b**). Data shown are representative of multiple replicates. *OS* osteogenic induction, *sh* short hairpin

**Fig. 7:**
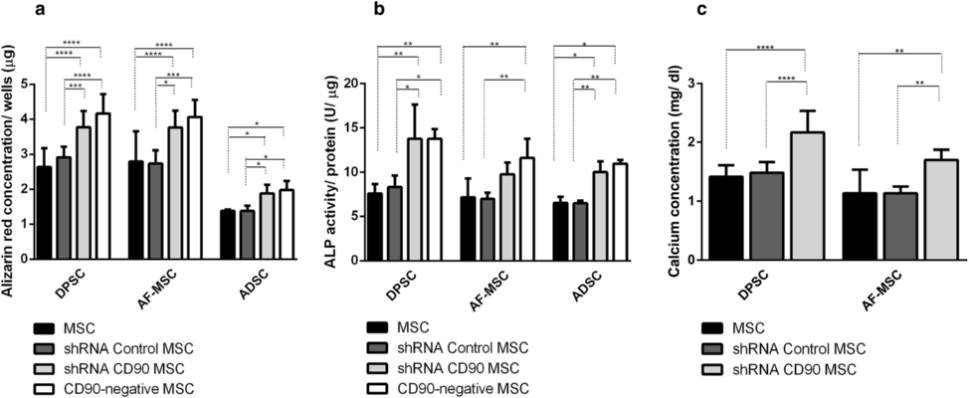
Quantitative evaluation of osteogenesis. **a** Quantification of Alizarin Red staining by dissolving the dye and subsequent absorption measurement. **b** Alkaline phosphatase (*ALP*) activity in cells cultured in osteogenic medium. ALP activity (mU μmol p-nitrophenol released per min) was normalized for protein. **c** Calcium concentration determinations were possible only for DPSC samples and AF-MSC samples. The data are expressed as mean ± SD and are representative of two independent experiments, each performed in triplicate (DPSC = 2 donors, ADSC = 2 donors, AF-MSC = 2 donors). *p < 0.05; **p < 0.01; ***p < 0.001. *ADSC* adipose tissue mesenchymal stromal cell, *AF-MSC* amniotic fluid mesenchymal stromal cell, *DPSC* dental pulp mesenchymal stromal cell, *MSC* mesenchymal stromal cell, *sh* short hairpin

The differentiation potentials of non-transduced MSCs, shRNA control MSCs, shRNA CD90 MSCs, and CD90-negative MSCs were analysed in parallel in multilineage (osteogenic and adipogenic) differentiation assays. MSCs isolated from dental pulp, amniotic fluid, and adipose tissue were submitted to osteogenic differentiation assays. As expected, osteogenic induction (OS) resulted in the occurrence of a mineralized matrix deposition which was detected 21 days after the initiation of differentiation induction. The mineralized matrix was assessed by: a) Alizarin Red S Staining (AR); b) determination of calcium concentration; and c) alkaline phosphatase activity. According to previous data reported by other groups [7, 59, 60], mineral deposition was higher in MSCs isolated from dental pulp than in those isolated from lipoaspirate tissue (Fig. 6). The AR staining pattern obtained differs according to the level of CD90 expression (Fig. 6). The shRNA CD90 MSCs showed significantly higher production of osteogenic matrices, with the visualization of a higher concentration of AR dye in the samples, in comparison to both non-transduced MSCs and shRNA control MSCs (Figs. 6 and 7a). Even higher mineralization was observed in CD90-negative MSC samples. The effect of reduced CD90 expression on the osteogenic differentiation of MSCs was also assessed by monitoring alkaline phosphatase activity, which demonstrated an enhanced production of this enzyme in cells with reduced CD90 expression (Fig. 7b). The calcium production by shRNA CD90 MSCs was also higher than in non-transduced MSCs (Fig. 7b). The calcium concentration could not be adequately measured in samples originating from lipoaspirate tissue due the low calcium concentration in all samples.

**Fig. 8:**
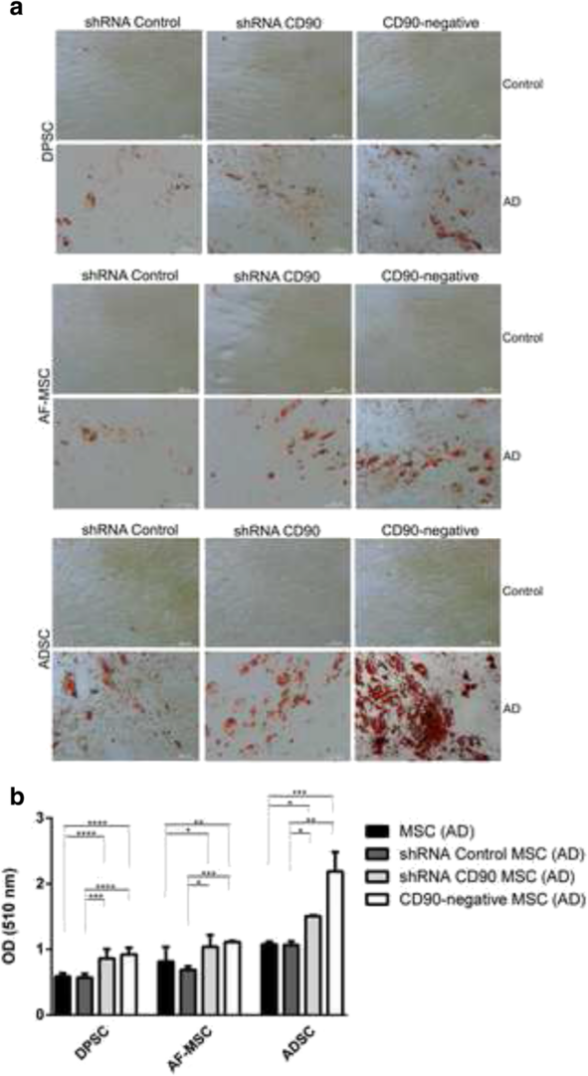
Reduction of CD90 expression stimulates the adipogenesis of mesenchymal stromal cells (*MSCs*). MSCs, short hairpin (sh)RNA control MSCs, shRNA CD90 MSCs, and CD90-negative MSCs were tested for their ability to differentiate into adipogenic lineages. **a** Representative photomicrograph images show oil red staining indicative of adipogenic differentiation. MSCs from dental pulp (*DPSC*), amniotic fluid (*AF-MSCs*), and lipoaspirate (*ADSC*) were cultured in the non-differentiation medium MSCs (control) and adipogenic differentiation medium (*AD*). The images shown are representative of two independent experiments. **b** Oil red dye retained in the lipid vacuoles was measured by determining the optical density (*OD*) at 510 nm. Data shown represent the mean ± SD of one experiment performed in triplicate (*n* = 7). *p < 0.05; **p < 0.01; ***p < 0.001

The adipogenic differentiation capacity of the MSCs was also analysed using MSCs isolated from dental pulp, adipose tissue, and amniotic fluid (Fig. 8). All cell populations showed significant morphological changes compared to those that were not incubated in adipogenesis-inducing medium. The cells presented an oval shape, with lipid vacuoles in the cytoplasm, and the presence of many lipid droplets as evidenced by Oil Red staining (Fig. 8a). We observed an increase in the number of adipocyte-like cells in shRNA CD90 MSCs compared to the shRNA control MSCs, with an even higher number of adipocyte-like cells in CD90-negative MSCs. Independent of CD90 expression, we found that MSCs from adipose tissue produced higher amounts of lipid droplets when compared to cells obtained from the amniotic fluid and dental pulp. The most prominent adipocyte formation, revealed by Oil Red staining, was observed in CD90-negative MSCs isolated from adipose tissue (Fig. 8b).

## Discussion

The biology of MSCs has been broadly studied [1, 22, 61, 62] because of their therapeutic potential. Therefore, we decided to study the function of CD90, one main immunophenotypical marker of MSCs, in order to better understand its relationship with MSC morphology, proliferation, and differentiation. Here, we used MSCs isolated from three sources (dental pulp, adipose tissue, and amniotic fluid) to verify whether the effects caused by CD90 ablation would be source-specific. We used lentivirus-mediated CD90-shRNAi to stably reduce CD90 expression and further evaluate its function in MSCs. Here, we generated MSC lines transduced with lentivirus-carrying small hairpins (shRNA) targeting CD90. After the establishment of CD90-shRNA expressing MSCs (in shRNA CD90 MSCs), the reduction of CD90 expression was confirmed in immunophenotypical analysis using flow cytometry (Fig. 1). We subsequently evaluated the immunophenotypic profiles of modified MSCs, in addition to CD90. As expected, we found that these cells expressed the positive MSC markers CD29, CD73, and CD105, and did not express the following cell markers: CD14, CD31, CD34, CD45, CD106, and HLA-DR (Additional file 2: Figure S1). Surprisingly, we found that a knockdown in CD90 expression in all CD90-shRNAi MSCs obtained here led to a reduction in the CD44 and CD166 expression (Fig. 5).

The role of CD166 in MSCs has not been determined to date. However, CD44 (hyaluronan receptor) [63] is expressed by a large number of cells and is involved in cell adhesion, migration, and homing in MSCs [64–66]. Furthermore, CD44 has been recognized as a stem cell marker for several types of cancer and is strongly linked to metastatic spread. It has been shown that the reduction of CD44 in cancer stem cells caused them to differentiate into non-cancer stem cells [67]. The receptor CD166 (activated leukocyte cell adhesion molecule, ALCAM) is a member of the immunoglobulin superfamily of cell adhesion molecules [68, 69] and is present in undifferentiated MSCs and other cell types [70]. Like CD44, CD166 has been shown to participate in tumour invasion [71–73]. A recent study using liver cancer cell lines relates the close interaction between CD44 and CD166. The authors showed that a knockdown of CD166 inhibits the expression of CD44 via the NFkB pathway [74].

CD90 has also been identified as a candidate marker for adult stem cells. Few studies have shown a functional association between CD90 and CD44 or CD166 markers. Strikingly, the few data showing association come from cancer stem cell research: CD90, CD44, and CD166 are notably considered cancer stem cell markers [44, 70, 71, 75]. Our results showed that the knockdown of CD90 leads to a decrease in CD44 and CD166 expression, which could indicate a shift in the stemness state of MSCs towards a state more susceptible to differentiation.

CD90 has been linked to the spindle-shape of lung fibroblasts. Observing lung fibroblasts sorted on the basis of CD90 expression, Phipps and co-workers [76] affirmed that the lung CD90^–^ fibroblast subpopulation showed a more polygonal shape than the spindle-shaped CD90^+^ fibroblasts. In contrast to these observations, in our study, a reduction in CD90 expression in shRNA CD90 MSCs and CD90-negative MSCs did not present altered morphology or proliferation rate when compared to control cells (Fig. 3). Here, we also demonstrated that a reduced expression of CD90 does not affect the immunosuppressive activity of MSCs on lymphocyte proliferation in vitro (Fig. 5), a very important therapeutic MSC property.

We carried out assays to investigate the differentiation of CD90-ablated MSCs into osteogenic and adipogenic lineages. In our differentiation assays, shRNA CD90 MSCs and CD90-negative MSCs showed a higher rate of adipogenic differentiation when compared to the controls (Fig. 8). In the same way, an enhanced osteogenic differentiation was observed in samples of shRNA CD90 MSCs and CD90-negative MSCs. Alizarin Red S staining showed that CD90-negative MSCs, the CD90 negative fraction of shRNA CD90 MSCs, accumulated more mineralized matrix than shRNA CD90 MSCs (Figs. 6 and 7). According to our results, the knockdown of CD90 expression in MSCs facilitates osteogenic and adipogenic differentiation.

Recently, Woeller and colleagues [77] showed that CD90 controls adipogenesis. They had previously observed that CD90-null mice gain weight at a faster rate, and that ectopic overexpression of CD90 blocked adipogenesis [77]. They also stated that, although pre-adipocyte fibroblasts expressed CD90, fat adipocytes presented almost undetectable CD90 levels. In agreement with this study, we also observed that a loss of CD90 expression in MSCs increased the production of adipogenic matrix in vitro. Based on their study, Woeller and colleagues [77] suggest that CD90 could be a new therapeutic target for obesity. However, our results indicate that this differentiation facilitation related to decreased CD90 expression is not only for adipogenic differentiation, since we observed the same facilitation for osteogenic differentiation. Our data indicate that the knockdown of CD90 seems to lower the stemness guard of MSCs, thereby enabling further differentiation when in the presence of the specific stimuli.

The finding that the level of CD90 regulates both MSC adipogenesis and osteogenesis is very interesting, because it is well accepted that differentiation stimuli usually cause an “inverse relationship” between adipogenic and osteogenic differentiation [78], although the molecular pathways that can converge into adipogenesis and osteogenesis have not been completely elucidated. Here, we demonstrated that the production of mineralized matrix directly correlates with the level of CD90 ablation: higher in the samples of CD90-negative MSCs than shRNA CD90 MSCs. It is unclear how CD90 can affect adipogenesis. However, it has been demonstrated that CD90 also regulates RhoGTPase activity in fibroblasts. Exogenous expression on CD90-non-expressing fibroblasts results in Rho GTPase activation [42]. CD90 participates in many signalling pathways, and it is becoming clear that, although CD90 has been recognized as a plain cell marker, it is also an important regulator of MSC signalling [79]. In order to accurately understand the effects of CD90 on all *cis*- and *trans*-signalling networks that it participates in, significant further studies are required. Improving our knowledge of these mechanisms may allow a better understanding of MSC stemness and differentiation.

An increasing number of studies have shown that MSCs from different sources display significantly diverse properties and characteristics that may impact on their future therapeutic applications. The capacity of differentiation may vary according to the cell source [7, 59, 60]. In agreement with previous reports [59, 80–82], we observed that cells from the dental pulp tissue and aminiotic fluid produced a larger quantity of osteogenic matrix than cells from adipose tissue (Figs. 6 and 7). Despite the expected variance in the differentiation potential among MSCs from different tissues [83, 84], we confirm that a reduction in CD90 expression leads to a more efficient osteogenic differentiation, irrespective of the source.

CD90 is a GPI-anchored protein expressed in various cell types. In general, it appears to influence cell proliferation, differentiation, migration, and survival. The functions of CD90 are tissue- and cell-specific and, in the present work, we found that shRNA-induced knockdown in human MSCs increases the differentiation efficiency of these cells. Our group previously showed that CD90 expression could be used as an indicator to follow the differentiation commitment degree of MSCs. Immediately after the induction of differentiation, a progressive decrease in CD90 mRNA level correlates with the degree of differentiation observed [25]. It is important to reiterate that the ablation of CD90 expression did not result in a spontaneous differentiation. However, it facilitated MSC differentiation in the presence of inductors, indicating that CD90 may play an important role in maintaining the undifferentiated state of MSCs, perhaps by acting as an obstacle to be overcome during the early steps of cellular differentiation commitment.

## Conclusions

Taken together, the current data indicate that the ablation of CD90 in MSCs represents a promising alternative strategy and an efficient approach to increase MSC differentiation efficiency in vitro; it may, therefore, be used in the future to improve MSC differentiation yields in cellular therapy. Further studies are needed to evaluate whether this approach facilitates all the in vitro differentiation protocols established for MSCs, and how the ablation of CD90 affects migration/homing and the therapeutic potential of those cells in in vivo MSC therapy models. Our results showed that the knockdown of CD90 leads to a decrease in CD44 and CD166 expression, which could indicate a shift in the stemness state of MSCs towards a state that is more susceptible to differentiation.

## Abbreviations

ALP, alkaline phosphatase; CD, cluster differentiation; CFSE, carboxyfluorescein succinimidyl ester; CFU-F, colony-forming unit—fibroblast; HLA-DR, human leukocyte antigen—antigen D related; MSC, mesenchymal stromal cell; PBS, phosphate-buffered saline; PHA, phytohaemagglutinin; shRNA, small hairpin RNA

## Additional files

Additional file 1: Table S1. Surface protein expression of transduced and non-transduced MSCs originated from dental pulp (*DPSC*) (*n* = 3), amniotic fluid (*AF-MSCs*) (*n* = 2), and lipoaspirate (*ADSC*) (*n* = 2) were analysed by flow cytometry. Data shown represent the mean MFI ± SD obtained in cytometry analysis performed in duplicate. (TIF 177 kb) Additional file 2: Figure S1. Representative flow cytometry data to characterise transduced and non-transduced MSC groups studied in this work. One representative immunophenotypic analysis of groups obtained from the same dental pulp tissue is shown. Unstained MSC (*grey shaded histogram*), MSC (*grey line*), shRNA control MSCs (*black slim line*), and shRNA CD90 MSCs (*black thick lines*) were harvested and labelled with Ab against CD90, CD44, CD166, CD73, CD29, CD14, CD45, CD31, CD34, CD106, and HLA-DR as indicated. FACS analysis demonstrated that MSCs and shRNA CD90 MSCs were negative for CD14, CD45, CD31, CD106, HLA-DR, and CD34, and were positive for CD105, CD73, and CD29. MSCs were positive for CD90, CD44, and CD166, whereas shRNA CD90 MSCs showed a reduction in CD90, CD44, and CD166 expression. (TIF 8631 kb)
