# Supplementary figures and images for: A reduction in CD90 (THY-1) expression results in increased differentiation of mesenchymal stromal cells
Source: Stem Cell Res Ther. 2016 Jul 28;7:97. doi: 10.1186/s13287-016-0359-3 (PMC4964048; doi:10.1186/s13287-016-0359-3)

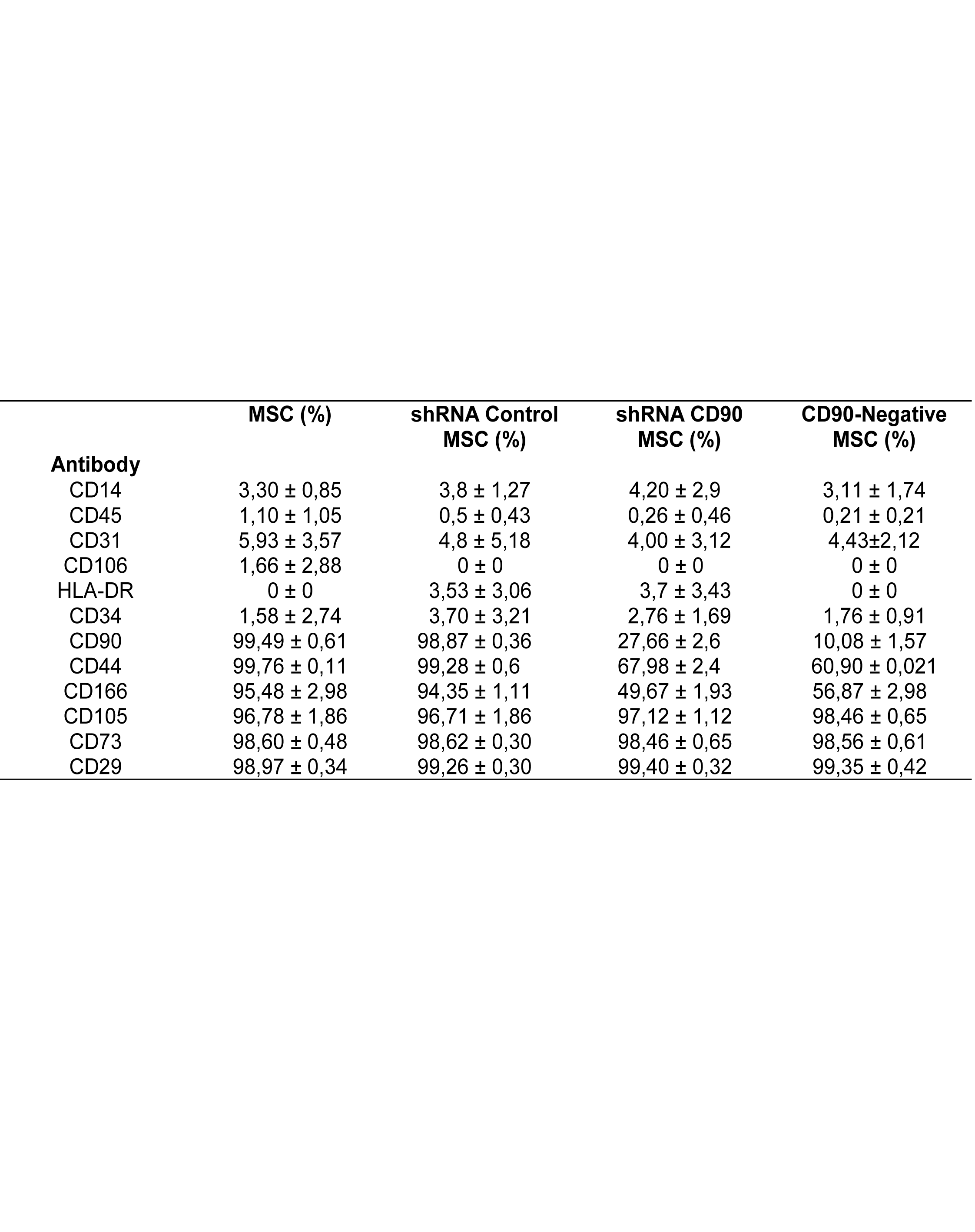

Supplement: Additional file 1: Table S1. — Surface protein expression of transduced and non-transduced MSCs originated from dental pulp (DPSC) (n = 3), amniotic fluid (AF-MSCs) (n = 2), and lipoaspirate (ADSC) (n = 2) were analysed by flow cytometry. Data shown represent the mean MFI ± SD obtained in cytometry analysis performed in duplicate. (TIF 177 kb) [file 13287_2016_359_MOESM1_ESM.tif]

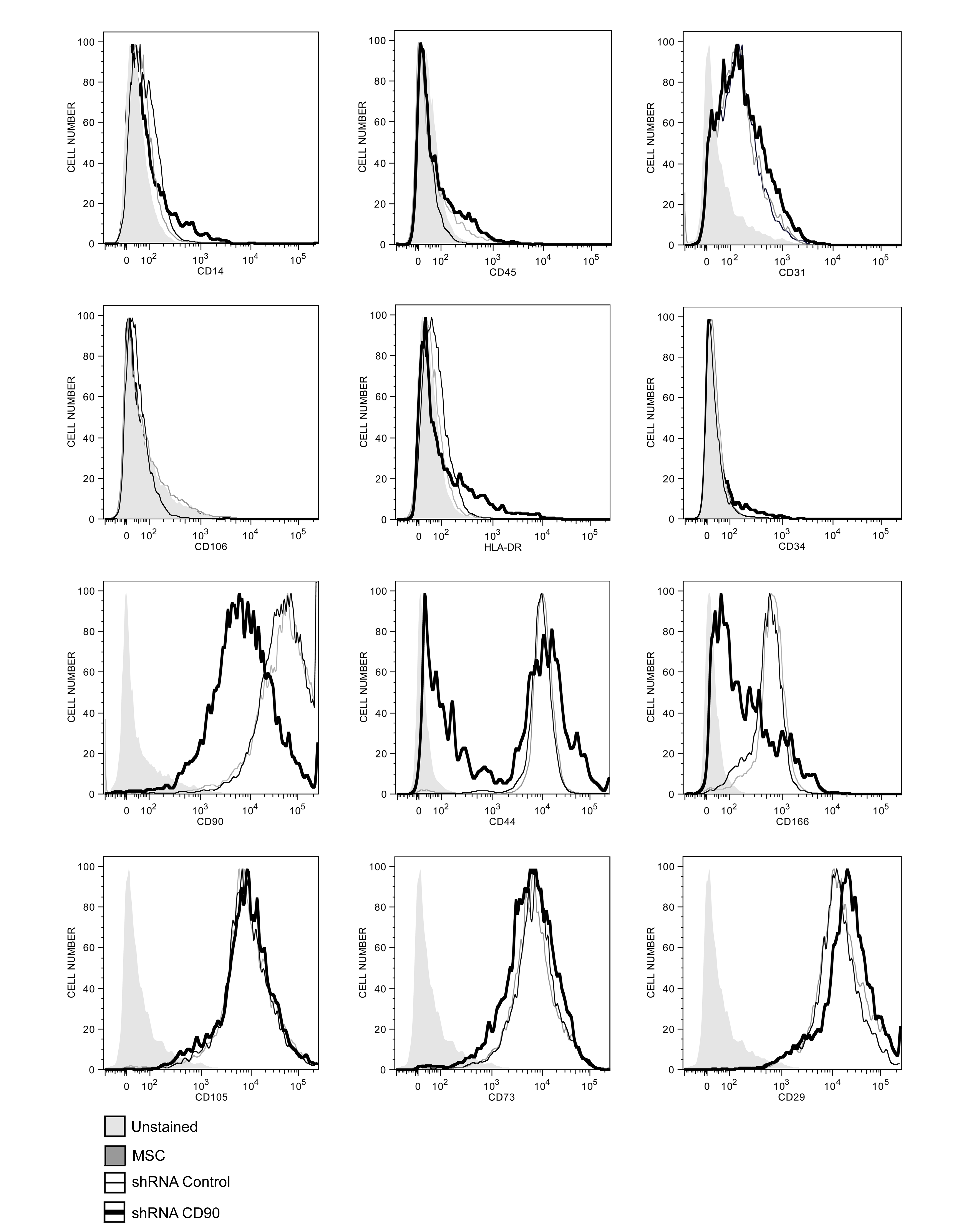

Supplement: Additional file 2: Figure S1. — Representative flow cytometry data to characterise transduced and non-transduced MSC groups studied in this work. One representative immunophenotypic analysis of groups obtained from the same dental pulp tissue is shown. Unstained MSC (grey shaded histogram), MSC (grey line), shRNA control MSCs (black slim line), and shRNA CD90 MSCs (black thick lines) were harvested and labelled with Ab against CD90, CD44, CD166, CD73, CD29, CD14, CD45, CD31, CD34, CD106, and HLA-DR as indicated. FACS analysis demonstrated that MSCs and shRNA CD90 MSCs were negative for CD14, CD45, CD31, CD106, HLA-DR, and CD34, and were positive for CD105, CD73, and CD29. MSCs were positive for CD90, CD44, and CD166, whereas shRNA CD90 MSCs showed a reduction in CD90, CD44, and CD166 expression. (TIF 8631 kb) [file 13287_2016_359_MOESM2_ESM.tif]
